# Correlation of microrna-372 upregulation with poor prognosis in human glioma

**DOI:** 10.1186/1746-1596-8-1

**Published:** 2013-01-08

**Authors:** Gang Li, Zhiguo Zhang, Yanyang Tu, Tianbo Jin, Hongjuan Liang, Guangbin Cui, Shiming He, Guodong Gao

**Affiliations:** 1 Department of Neurosurgery, Tangdu hospital, the Fourth Military Medical University, No. 569, Xinsi Road, Xi’an, 710038, China; 2 Department of Clinical Experimental Surgery, Tangdu hospital, the Fourth Military Medical University, Xi’an, 710038, China; 3 National Engineering Research Center for Miniaturized Detection Systems, School of Life Sciences, Northwest University, Xi’an, 710069, China; 4 Department of Radiology, Tangdu hospital, the Fourth Military Medical University, Xi’an, 710038, China

**Keywords:** miR-372, Glioma, Real-time quantitative RT-PCR assay, Prognosis

## Abstract

MicroRNA-372 (miR-372) acts as either an oncogenic miRNA or an anti-oncomiR in various human malignancies. However, its roles in gliomas have not been elucidated. To address this problem, we here detected miR-372 expression in human gliomas and non-neoplastic brain tissues by real-time quantitative RT-PCR assay. The association of miR-372 expression with clinicopathological factors or prognosis of glioma patients was also statistically analyzed. As the results, miR-372 expression levels were significantly upregulated in glioma tissues compared to the corresponding non-neoplastic brain tissues (P<0.001). In addition, the high miR-372 expression was significantly associated with the advanced pathological grade (P=0.008) and the low Karnofsky performance score (KPS) of glioma patients (P=0.01). Moreover, the overall survival of patients with high miR-372 expression was dramatically shorter than those with low miR-372 expression (P<0.001). Furthermore, multivariate Cox regression analysis indicated that miR-372 expression was an independent prognostic factor for glioma patients (P=0.008). More importantly, subgroup analyses according to tumor pathological grade revealed that the cumulative overall survival of glioma patients with advanced pathological grades was significantly worse for high miR-372 expression group than for low miR-372 expression group (P<0.001), but no significant difference was found for patients with low pathological grades (P=0.08). Taken together, these data offer the convincing evidence for the first time that miR-372 may act as an oncogenic miRNA in gliomas and represent a potential regulator of aggressive development and a candidate prognostic marker for this malignancy, especially for advanced tumors with high pathological grades.

**Virtual slides:**

The virtual slide(s) for this article can be found here: http://www.diagnosticpathology.diagnomx.eu/vs/1707761328850011

## Introduction

Human gliomas are a heterogeneous group of primary intracranial tumors for both children and adults [1]. The entities are distinguished based on morphological criteria by histological analysis and presumed cell of origin. According to the World Health Organization (WHO) classification which is based on histomorphological criteria, human gliomas includes well-differentiated low grade astrocytomas [World Health Organization (WHO) grade I~II], anaplastic astrocytomas (WHO grade III) and glioblastoma multiforme (GBM, WHO grade IV) [2]. Despite great progress in therapeutic technologies, such as surgery, radiotherapy, photodynamic therapy, and chemotherapy, the clinical outcome of patients with gliomas remains poor, with a lower than 3% 5-year survival rate for patients with GBM [3]. Although the WHO classification can reflect the anticipated malignancy of the tumor and serve as a criterion to predict the clinical outcome of patients, recent studies have indicated that histomorphological criteria alone may not be sufficient to estimate the prognosis [2,4-8]. For example, Curran et al. [9] demonstrated that the median survival time of patients with high-grade gliomas range from 5 to 59 months and some patients with low-grade tumors also present poor outcome. Therefore, to investigate the molecular genetics of gliomas may help to overcome some of these limitations.

MicroRNAs (miRNAs) are a recently discovered class of short non-coding endogenous RNA molecules that have a wide impact on the regulation of multiple target genes’ expression post-transcriptionally [10]. At first, miRNAs are transcribed by RNA polymerase II to yield long transcripts known as pri-miRNAs, which are processed to pre-miRNAs by the RNase III enzyme Drosha in the nucleus; then, the pre-miRNAs are exported to the cytoplasm by exportin-5 and subsequently converted to mature duplex miRNAs by another RNase III enzyme, Dicer; after that, mature miRNAs regulate their targets by direct cleavage of the mRNA or by inhibition of protein synthesis, according to the degree of complementarities with their targets’ 3^′^UTR regions [11,12]. With the use of sophisticated techniques and screening tools, miRNAs have been demonstrated to be involved in multiple cellular processes, including development, cell proliferation and differentiation, stem cell maintenance, epithelial- mesenchymal transition, apoptosis and metabolism [13,14]. miRNAs also play important roles in a wide variety of physiological and pathological processes involved in tumorigenesis and tumor progression. Depending on their target genes, miRNAs can function either as oncogenes or tumor suppressors. Accumulating findings have demonstrated that miRNAs are associated with glioma formation and growth [15,16]. In the present study, we focus on miR-372, which has been demonstrated to act as either an oncogenic miRNA or an anti-oncomiR in various human malignancies [7,17,18]. However, its roles in gliomas have not been elucidated. To address this problem, miR-372 expression in human gliomas and non-neoplastic brain tissues was measured by real-time quantitative RT-PCR assay. The association of miR-372 with clinicopathological factors or prognosis of glioma patients was also statistically analyzed.

## Materials and methods

### Patients and tissue samples

This study was approved by the Research Ethics Committee of Tangdu Hospital, Fourth Military Medical University, P. R. China. Written informed consent was obtained from all of the patients. All specimens were handled and made anonymous according to the ethical and legal standards.

One hundred and twenty-eight pairs of glioma and adjacent non-neoplastic brain tissues resected between 2000 and 2010 were retrieved from the archives of the Pathology Department of Tangdu Hospital, Fourth Military Medical University, P. R. China. All the slides of glioma tissues were re-evaluated according to WHO classifications [2] by two pathologists, with differences resolved by careful discussion. A total of 76 males and 52 females (1.46:1) were enrolled in this study, and the median age was 42 years (range, 12–71). Thirty-two of the 128 gliomas were classified as low-grade [18 pilocytic astrocytomas (WHO I) and 14 diffuse astrocytomas (WHO II)], and 96 were classified as high-grade gliomas [38 anaplasia astrocytomas (WHO III), and 58 primary glioblastomas (WHO IV)]. None of the patients had received chemotherapy or radiotherapy prior to surgery. All the tissues were snap-frozen in liquid nitrogen and stored at −80°C following surgery for real-time quantitative RT-PCR assay. The clinicopathological features and the treatment strategies of all the patients were indicated in Table 1.

**Table 1:** Clinicopathological features of 128 patients with gliomas

**Features**	**WHO I**	**WHO II**	**WHO III**	**WHO IV**
**Case No.**	18	14	38	58
**Mean age (year)**	38.6	45.9	43.1	44.2
**Gender**				
Male	12	6	25	33
Female	6	8	13	25
**KPS**				
>80	15	11	9	15
<80	3	3	29	43
**Surgery**				
Gross total resection	18	14	28	38
Partial resection	0	0	9	15
Biopsy	0	0	1	5
**Adjuvant treatment**				
Radiotherapy	0	0	30	12
Chemotherapy	0	1	0	6
Radiotherapy and Chemotherapy combination	0	0	5	28

Clinical follow-up was available for all patients (median, 16 months; range, 1–148 months). Follow-up information for all patients was obtained every 3 months by telephone, at a visit or via a posted questionnaire. During the follow-up period, overall survival was measured from diagnosis to death or the last follow-up (5 years). Patients, who died of diseases not directly related to their gliomas or due to unexpected events, were excluded from this study.

### Real-time quantitative RT-PCR for miRNA

The expression of miR-372 in glioma and adjacent non-neoplastic brain tissues was measured by real-time quantitative RT-PCR analysis according to the conventional protocols of Tangdu hospital [19]. Briefly, total RNA was extracted from frozen samples using Trizol reagent (Invitrogen, Shanghai, China) according to the users’ instruction. RNA concentration and purity were measured using the NanoDrop ND-1000 spectrophotometer (NanoDrop Technologies, Houston, TX, USA). Only the samples with the OD A260/A280 ratio close to value of 2.0, which indicates that the RNA is pure, were subsequently analyzed. The miR-372 and RNU6B (as an internal control)-specific cDNA were synthesized from total RNA using gene-specific primers according to the TaqMan MicroRNA assays protocol (Applied Biosystems, Foster City, CA, USA). Each reaction included 1×primer probe mix (TaqMan; ABI), 1× universal PCR master mix (TaqMan; ABI), and 200 ng of cDNA. Relative quantification of target miRNA expression was evaluated using the comparative cycle threshold (CT) method. Each sample was examined in triplicate and the raw data were presented as the relative quantity of target miRNA, normalized with respect to RNU6B.

### Statistical analysis

All computations were carried out using the software of SPSS version13.0 for Windows (SPSS Inc, IL, USA). Data were expressed as means±standard deviation (SD). Paired samples T test has been performed to compare the expression levels of miR-372 between glioma and paired non-neoplastic brain tissues. The analysis of variance (ANOVA) was used to determine the statistical differences among the groups. A life table was calculated according to the Kaplan-Meier method. Hazard ratios for the time-to-event endpoint were estimated using the multivariate Cox regression analysis in a forward stepwise method to evaluate the effect of multiple independent prognostic factors on survival outcome. Differences were considered statistically significant when *p* was less than 0.05.

## Results

### miR-372 upregulation in human glioma tissues

**Figure 1:**
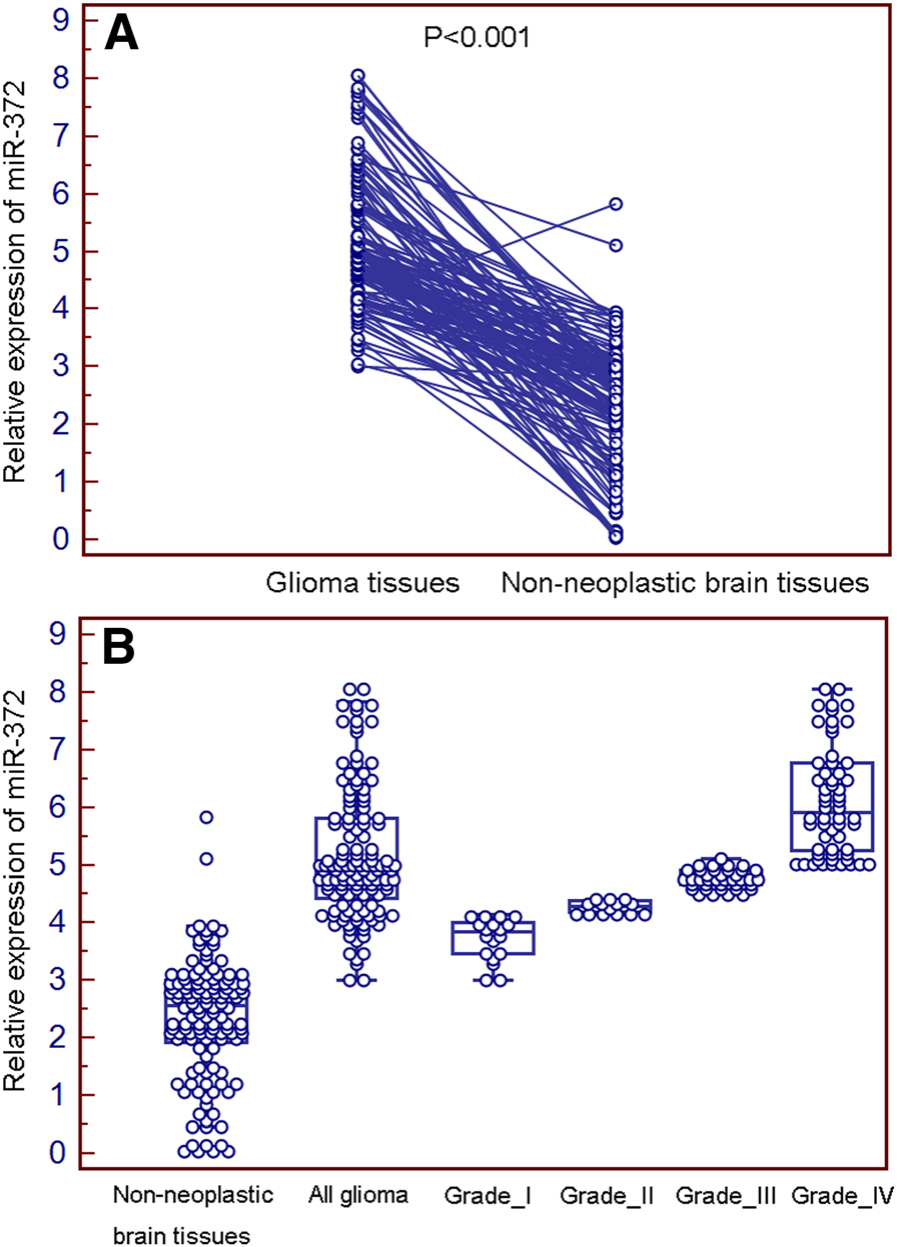
**miR-372 expression in 128 pairs of glioma and adjacent non-neoplatic brain tissues detected by quantitative real-time polymerase chain reaction (qRT-PCR) analysis.** (**A**) Expression levels of miR-372 in glioma and paired non-neoplastic brain tissues. (**B**) Expression levels of miR-372 in non-neoplastic brain tissues and glioma tissues with different pathological grades (Grade I~IV).

MiR-372 expression was detected in 128 pairs of glioma and adjacent non-neoplastic brain tissues normalized to RNU6B. As shown in Figure 1A, we found that the expression of miR-372 was distinctly increased in glioma tissues compared to non-neoplastic brain tissues (mean±SD: 5.2±1.1 vs. 2.4±1.1, P<0.001). In addition, miR-372 expression in high-grade (III-IV; 5.6±1.0) and low-grade (I-II; 3.9±0.4) gliomas were both significantly higher than that in non-neoplastic brain tissues (2.4±1.1; P<0.001 and 0.001, respectively, Figure 1B). There was also a significant difference in miR-372 expression between high-grade (III-IV) and low -grade (I-II) glioma tissue specimens (P=0.001, Figure 1B).

### MiR-372 upregulation associates with advanced clinicopathological features of gliomas

We then analyzed the association between miR-372 expression and clinicopathological parameters in gliomas. Glioma tissues expressing miR-372 at levels less than the median expression level (4.9) were assigned to the low expression group (mean expression value 4.3, n=50), and those samples with expression above the median value were assigned to the high expression group (mean expression value 5.8, n=78). The high level of miR-372 expression was significantly more common in glioma tissues with advanced pathologic grade than those with low pathologic grade (P=0.008, Table 2). A significant relationship was also observed between miR-372 expression and the KPS. miR-372 upregulation occurred more frequently in tumors with low KPS than those with high KPS (P=0.01). No significant association was found between miR-372 expression and gender or age at diagnosis.

**Table 2:** Association of miR-372 expression in human glioma tissues with different clinicopathological features

**Clinicopathological features**	**No. of cases**	**miR-372 expression**		**P**
		**High (n, %)**	**Low (n, %)**	
**WHO grade**				
I	18	2 (11.1)	16 (88.9)	0.008
II	14	2 (14.3)	12 (85.7)	
III	38	24 (63.2)	14 (36.8)	
IV	58	50 (86.2)	8 (13.8)	
**Age**				
<55	52	33 (63.5)	19 (36.5)	NS
≥55	76	45 (59.2)	31 (40.8)	
**Gender**				
Male	76	45 (59.2)	31 (40.8)	NS
Female	52	33 (63.5)	19 (36.5)	
**KPS**				
<80	78	56 (71.8)	22 (28.2)	0.01
≥80	50	22 (44.0)	28 (56.0)	

### Relationship of miR-372 expression with overall survival in patients with gliomas

**Figure 2:**
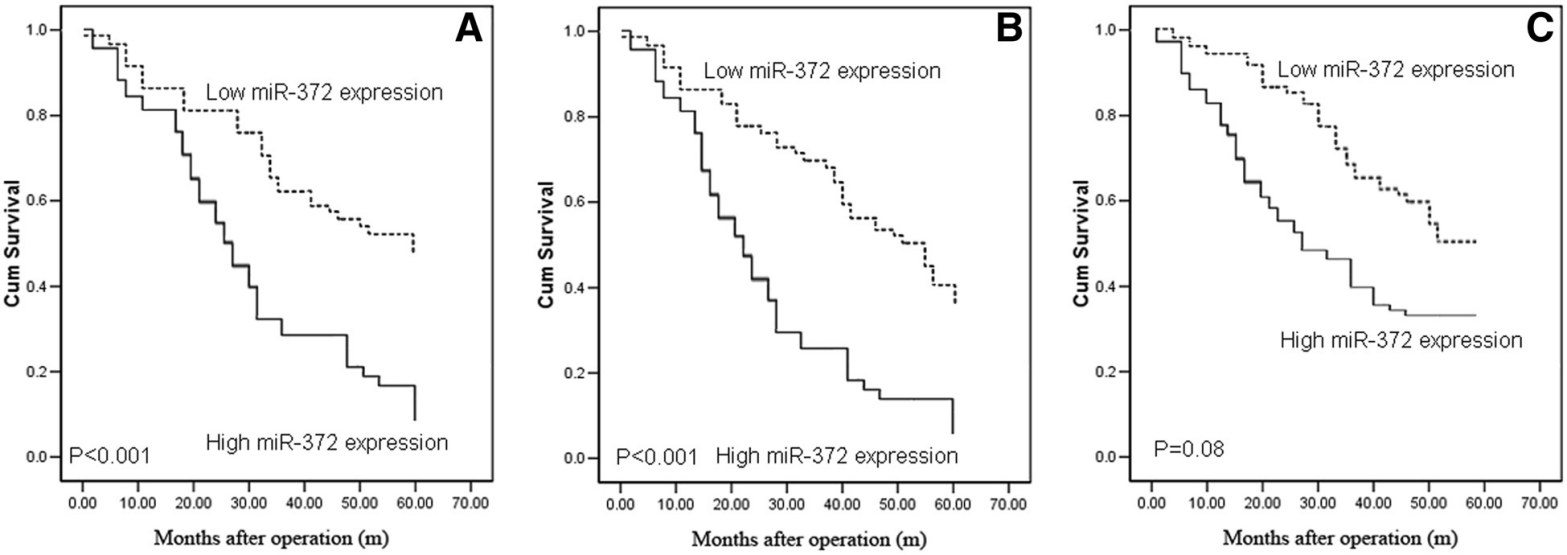
**Kaplan-Meier survival curves for glioma patients with high or low expression of miR-372.** (**A**) The 5-year overall survival rate of all 128 glioma patients with high or low miR-372 expression; (**B**) The 5-year overall survival rate of 78 glioma patients with advanced pathological grades (Grade III~IV) in high or low miR-372 expression group; (**C**) The 5-year overall survival rate of 50 glioma patients with low pathological grades (Grade I~II) in high or low miR-372 expression group.

In order to investigate the relationship between miR-372 expression and clinical outcome in gliomas, the clinical information of the glioma patients in miR-372-high or -low groups was reviewed. During the follow-up period, 100 of 128 glioma patients (78.1%) had died [72 (92.3%) from the miR-372-high group and 28 (56.0%) from the miR-372-low group]. As determined by the log-rank test, the survival rate of patients with high miR-372 expression was significantly lower than those with low miR-372 expression (P<0.001; Figure 2). In multivariate analysis, Cox proportional hazards model involving the expression level of miR-372 protein and various clinical parameters identified miR-372 upregulation (P=0.008) as an independent prognostic factor for glioma patients. Statistical values of the expression of miR-372 and other clinical parameters derived from Cox stepwise proportional hazards model were indicated in Table 3.

**Table 3:** Cox multivariate analysis

**Parameter**	**Risk ratio**	**95% confidence interval**	**P**
**Age**	0.89	0.58-1.65	0.71
**Gender**	1.02	0.66-1.83	0.33
**KPS**	1.99	1.28-2.95	0.06
**Extent of resection**	1.29	0.89-2.13	0.11
**Type of adjuvant treatment**	1.37	1.02-2.24	0.11
**miR-372 expression**	4.37	2.11-8.93	0.008

More importantly, subgroup analyses according to tumor pathological grade revealed that the cumulative overall survival of glioma patients with advanced pathological grade (Grade III~IV) was significantly worse for high miR-372 expression group than for low miR-372 expression group (P<0.001, Figure 2B), but no significant difference was found for patients with low pathological grades (Grade I~II, P=0.08, Figure 2C).

## Discussion

Biomarker screening is an emerging field for neurooncology. Especially for gliomas, considerable progresses have been made in identifying, characterizing, and applying molecular markers. In the present study, we initially found that miR-372 was upregulated in human glioma tissues compared with non-neoplastic brain tissues. Then, the increased expression of miR-372 in glioma tissues was significantly correlated with advanced tumor progression and aggressive clinicopathological features. Next, the Kaplan-Meier analysis revealed that glioma patients with high miR-372 expression tend to have poorer overall survival. In addition, the multivariate analysis clearly demonstrated that high miR-372 expression was a statistically significant risk factor affecting overall survival in glioma patients, suggesting that miR-372 upregulation in gliomas is not only in a grade-dependent fashion, it is also a predictor of overall survival. Finally, subgroup analyses showed the significant prognostic value of miR-372 upregulation for glioma patients especially for those with advanced pathological grade.

MiR-372, together with miR-371a, miR-371b and miR-373, belongs to miR-371~373 cluster which has been demonstrated to play important roles in tumorigenesis and tumor progression [20]. Among these members, miR-372 may act as either an oncogenic miRNA or an anti-oncomiR in various human malignancies. It can enhance cell proliferation, stimulate cell cycle progression, and decrease apoptosis of tumor cells in many types of cancer. For example, Cho et al. [17] revealed that miR-372 plays an oncogenic role through down-regulation of the tumor suppressor gene LATS2, which accelerated growth and survival of gastric cancer cells; Yamashita et al. [21] indicated that the increased expression of miR-372 in colon cancer was an independent prognostic factor and was associated with synchronous liver metastasis; Voorhoeve et al. [14] demonstrated that miR-372 could enhance cell proliferation, stimulate cell cycle progression, or decrease apoptosis in testicular germ cell tumors. Consistent with these previous studies, our data also found the upregulation of miR-372 in glioma tissues compared with paired adjacent non-neoplastic brain tissues. In addition, the aberrant expression of miR-372 was associated with advanced pathological grades and low KPS of glioma patients, indicating that this miRNA may be involved in the development of human gliomas. By contrast, accumulating studies showed the tumor suppressive roles of miR-372 in many cancers. For example, Tian et al. [22] found the downregulation of miR-372 in cervical carcinoma tissues as compared with adjacent normal cervical tissues. The authors demonstrated that its anti-oncogenic role might be through control of cell growth and cell cycle progression by down-regulating the cell cycle genes CDK2 and cyclin A1. These findings suggest that the different expression patterns and involvement of miR-372 in various cancers may depend on the roles of its target genes.

Our findings that miR-372 upregulation was associated with aggressive tumor progression mentioned above prompt us to investigate its possible prognostic value in glioma patients. According to the univariate and multivariate analyses, we identified miR-372 upregulation as an independent predictor for short overall survival of glioma patients, which was consistent with the findings of Yamashita et al. [21] in colon cancer. Interestingly, our subgroup analyses further suggested that miR-372 may act as a significant prognostic factor for glioma patients with high pathological grades (III~IV), but not for those with low pathological grades (I~II).

## Conclusion

In conclusion, our data offer the convincing evidence for the first time that miR-372 may act as an oncogenic miRNA in gliomas and represent a potential regulator of aggressive development and a candidate prognostic marker for this malignancy, especially for advanced tumors with high pathological grades. Further elucidation of the mechanism by which the oncogenic roles of miR-372 in gliomas are thwarted is worth to be done.

## Competing interests

The authors declare that they have no competing interests.

## Authors’ contributions

SH and GG designed the study. GL and ZZ participated in the design and coordination, performed the molecular genetic evaluation, and drafted the manuscript. All the patients were followed up by YT and HL. And TJ and GC performed the statistical analysis, and joined into drafting the manuscript. GL, SH and GG all contributed to improving the draft of the manuscript. All authors have read and approved the final manuscript.
